# Determination of inflammatory biomarkers in patients with COPD: a comparison of different assays

**DOI:** 10.1186/1471-2288-12-40

**Published:** 2012-03-31

**Authors:** José L López-Campos, Elena Arellano, Carmen Calero, Ana Delgado, Eduardo Márquez, Pilar Cejudo, Francisco Ortega, Francisco Rodríguez-Panadero, Ana Montes-Worboys

**Affiliations:** 1 Unidad Médico-Quirúrgica de Enfermedades Respiratorias, Instituto de Biomedicina de Sevilla (IBiS), Hospital Universitario Virgen del Rocío, Sevilla, Spain; 2 CIBER de Enfermedades Respiratorias (CIBERES), Sevilla, Spain; 3 Departamento de Bioquímica, Hospital Universitario Virgen del Rocío, Sevilla, Spain

## Abstract

**Background:**

Chronic obstructive pulmonary disease (COPD) is an inflammatory pulmonary disorder with systemic inflammatory manifestations that are mediated by circulating acute-phase reactants. This study compared an enzyme-linked immunosorbent assay (ELISA) to a nephelometric technique for the measurement of serum C-reactive protein (CRP) and serum amyloid A (SAA) and investigated how the choice of assay influenced the estimation of inflammation in patients with stable COPD.

**Methods:**

CRP and SAA concentrations measured by ELISA and nephelometry in 88 patients with COPD and 45 control subjects were used to evaluate the performance of these methods in a clinical setting.

**Results:**

With both assays, the concentrations of CRP and SAA were higher in COPD patients than in controls after adjustment for age and sex. There was a moderate correlation between the values measured by ELISA and those measured by nephelometry (logCRP: r = 0.55, p < 0.001; logSAA: r = 0.40, p < 0.001). However, the concentrations of biomarkers determined by nephelometry were significantly higher than those obtained with ELISA for CRP (mean difference = 2.7 (9.4) mg/L) and SAA (mean difference = 0.31 (14.3) mg/L).

**Conclusion:**

Although the serum CRP and SAA concentrations measured by ELISA and nephelometry correlated well in COPD patients, the ELISA values tended to be lower for CRP and SAA when compared with nephelometric measurements. International standardization of commercial kits is required before the predictive validity of inflammatory markers for patients with COPD can be effectively assessed in clinical practice.

## Background

Chronic obstructive pulmonary disease (COPD) is a complex chronic inflammatory disease of the lungs with significant extrapulmonary effects that may contribute to its severity in individual patients [1,2]. Growing evidence suggests that markers of systemic inflammation, such as C-reactive protein (CRP) and serum amyloid A (SAA), are increased in patients with COPD compared with control subjects without COPD [3]. For this reason, low-grade systemic inflammation is currently considered to be a hallmark of COPD and one of the key mechanisms that may be responsible for the increased rate of comorbidity.

CRP is the prototypic acute-phase reactant that belongs to the highly conserved pentraxin family of plasma proteins. Current evidence indicates that increased CRP levels can be used to identify subjects who have an increased risk of developing myocardial infarction, stroke, unstable angina, or sudden cardiac death [4]. Elevated levels of CRP in patients with COPD were demonstrated to predict adverse outcomes and the development of cardiovascular complications [5]. In recent years, CRP has emerged as a biochemical marker of systemic involvement in COPD [6], a prognostic factor [7], and a marker for diagnosis and prognosis during acute exacerbations [8].

SAA is another acute-phase protein that is expressed primarily in the liver as a part of the systemic response to various injuries and inflammatory stimuli [9]; SAA levels rise with systemic inflammation in a manner similar to that of CRP [10]. Previous studies have suggested that SAA levels are increased in patients with COPD exacerbations [11,12] and in those with stable disease [13].

Although many studies of COPD have reported changes in various inflammatory markers, their relevance to the systemic manifestations of the disease is still unclear and is probably not causal [14]. In this regard, many questions concerning the selection of appropriate cut-off values for clinical use, the exact source of different inflammatory markers, and the impact of these markers on the systemic manifestations of COPD remain unanswered [15]. An important caveat is the current lack of standardization of the various analytical assays. Several analytical techniques, including enzyme-linked immunosorbent assays (ELISA) [13], nephelometry [12], chemiluminescent assays [6], and latex-enhanced immunoturbidimetric assays [16], have been developed to measure serum levels of inflammatory markers. A comparison of methods is usually recommended when two or more analytical assays are available in clinical practice. Because the measurements of inflammatory markers in patients with COPD are intended for clinical use, a difference of any given measurement method from another is clinically acceptable only when the diagnostic or prognostic aspects are not impaired. In this context, the question arises whether the laboratory technique used to measure serum concentrations of CRP and SAA can affect the interpretation of the results.

In the present study, we sought to compare an ELISA assay to a nephelometric technique for the measurement of CRP and SAA in patients with stable COPD and to investigate how the assay choice can influence estimates of inflammation in this group of patients.

## Methods

### Participants

This case-control study was conducted at the University Hospital Virgen del Rocio, Seville, Spain. All analyses were conducted in a cross-sectional fashion. Recruitment of cases and controls (2:1 ratio) occurred between May 2007 and November 2009 in our outpatient pulmonary clinic. Ethical approval for the study was granted by the local Institutional Ethics Committee (Comité de Ética e Investigación Clínica del Hospital Universitario Virgen del Rocío, Seville, Spain), and written informed consent was obtained from all participants. The inclusion criteria for the cases comprised the following: (a) a diagnosis of COPD with a forced expiratory volume in 1 second (FEV_1_)/forced vital capacity (FVC) ratio < 0.7 and (b) a negative history of acute exacerbations in the previous three months. Control subjects were recruited among persons who attended our tobacco weaning clinic. Smokers aged > 40 years with a FEV_1_/FVC ratio ≥ 0.7 were considered eligible for inclusion as controls. In all participants, the smoking status was confirmed by exhaled carbon monoxide measurement.

The exclusion criteria for cases and controls included a previous history of ischemic heart disease, congestive heart failure, ventilator dependency, malignancy, hepatic cirrhosis, end-stage renal disease, rheumatologic disorders, tuberculosis, orthopedic conditions precluding performance in the walking and cardiopulmonary exercise tests, neurological or psychiatric illnesses that could interfere with the participation in the study, or any systemic infection or inflammatory process that could be associated with increased CRP or SAA concentrations. All participants underwent a cardiopulmonary exercise test to rule out the presence of ischemic heart disease. If the test was positive, the subject was excluded from the study and referred to the cardiology department for appropriate care.

### Clinical assessment

To characterize the sample, one author (JLLC) assessed all of the participants using a standardized questionnaire that included questions on the presence of comorbidities, respiratory symptoms, and current medication use. The patients' anthropometric measures were collected in a standard fashion. A detailed medical history was obtained from all participants. Comorbidities were determined using the Charlson-age index [17]. All patients underwent spirometric tests according to the international standards [18]. Functional dyspnea was scored using the modified Medical Research Council scale [19].

Exercise testing was performed on a cycle ergometer MasterScreen CPX (ViaSys Healthcare, Hoechberg, Germany) while breathing room air. The protocol consisted of the following steps: 3 minutes of rest and 1 minute of no-load work, followed by a 15 Watts/min exercise routine with continuous 12 lead electrocardiogram (ECG) monitoring. The patients exercised until exhaustion or evidence of ischemic heart disease based on clinical criteria or the ECG results. The ECG was interpreted by an independent physician to assess the presence of exercise-induced myocardial ischemia. Minute ventilation, oxygen uptake, and carbon dioxide output were measured breath by breath.

### Laboratory methods

Blood samples were drawn by venipuncture before any test was performed, after the subjects had rested. The samples were stored in a -70°C freezer until measurement. Serum CRP (R&D System, Minneapolis, MN, USA) and SAA (Anogen, Mississauga, Ontario, Canada) concentrations were measured using commercially available high-sensitivity ELISA assays. Both biomarkers were also measured by immunonephelometry (Ne) (Dade Behring, Marbrug, Germany) according to the manufacturer's instructions. The use of both measurement methods in this study was justified by the availability of both techniques in our institute. In addition, we have our own experienced personnel dedicated to their determination. The minimum detection limits of the nephelometric assay were 0.16 mg/L and 0.28 mg/L for CRP and SAA, respectively. The minimum detection limits of the ELISA assays for CRP and SAA were 10^-4 ^mg/L and 0.011 mg/L, respectively. All samples were identified by a numerical code and analyzed in a random order. These assays were performed by an investigator who was blinded to the sources of the samples. Each sample was analyzed and duplicated, and the mean value of the two measures was used for the analyses.

ELISA measurements of CRP were performed in a final volume of 50 μL using 100-fold diluted serum samples. Samples were added to a 96-well microtiter plate that was pre-coated with a mouse monoclonal antibody specific to CRP and then incubated for 2 h. After four washes, the sera were incubated for 2 h with horseradish peroxidase (HRP)-conjugated anti-CRP antibodies for detection. The plates were washed four times, the substrate solution was then added, and the plates were incubated for 30 min. Finally, 50 μL of stop solution was added to each well, and the color changed from blue to yellow. The optical density of each well was measured at 450 nm using a microplate reader (Tecan, Männedorf, Switzerland). The results were linearized by plotting the log of the CRP concentration versus the log of the optical density, and the best fit line was determined by regression analysis. Because the samples were previously diluted, the concentrations read from the standard curve were corrected by the dilution factor.

SAA was determined by ELISA following the recommended protocol. Serum samples were diluted twice to a final dilution of 1:50. Aliquots (100 μL) of each sample were added to a microtiter plate that was pre-coated with a monoclonal antibody specific for SAA and incubated for 1 h. After five washes, a horseradish peroxidase-conjugated polyclonal antibody specific for SAA was added for 1 h. The plate was washed five times, and the substrate solution was added for 15 min. Finally, a stop solution was added, and the optical density was read at 450 nm using a microplate reader (Tecan, Männedorf, Switzerland). The standard curve provided in the kit was used to determine the amount of SAA in the unknown samples. To determine the final concentration of SAA serum, the concentration read from the standard curve was multiplied by the dilution factor.

The nephelometric determinations of CRP were performed using a polystyrene-enhanced immunonephelometric method on a Dimension Vista System (Siemens, Munich, Germany). Commercially available kits were used (Dade Behring, Marburg, Germany). The nephelometric determinations of SAA were performed using a latex-enhanced immunonephelometric method on a Dade Behring BN2 Nephelometer Analyzer equipped with commercially available kits (Dade Behring, Marburg, Germany).

### Statistical analysis

All statistical analyses were performed using Predictive Analytics Software, version 18.0 (PASW; IBM Corporation, Somers, New York, USA). The normality of the distribution was checked for all continuous variables; skewed data were log-transformed before further analysis. Normally distributed variables are expressed as arithmetic means with standard deviations in parentheses. Qualitative variables are summarized as absolute and relative frequencies. The reference values for each marker in the study cohort were determined by calculating the mean and the 95% confidence interval in the control group for each technique. Differences in continuous variables were assessed using Student's *t *test for independent samples (including Levene's test for variance similitude analysis). The χ^2 ^test and Fisher's exact test were used for categorical variables. The association of CRP and SAA values between cases and controls was assessed using Student's *t *test for independent samples (including Levene's test for variance similitude analysis). This association was further tested using a binomial multiple logistic regression analysis for which sex and age were included as covariates. The results of this analysis are expressed as adjusted odds ratios (ORs) with 95% confidence intervals (CIs).

We also investigated the differences between the two analytical methods for the measurements of CRP and SAA. First, we compared the mean values obtained via the two techniques using a paired Student's *t *test. Second, we performed a simple linear correlation analysis using Pearson's correlation coefficient. The degree of correlation was evaluated using the criteria of Brennan and Silman [20]. We then tested the degree of agreement between the two analytical methods using the Bland-Altman plot of difference versus mean [21]. Because no universally accepted cut-off values for CRP and SAA exist, we considered the average value of the mean concentrations that were obtained using the two techniques in the control group as a reference. We then calculated the number of patients who had levels of each inflammatory marker above the reference value for each technique and subsequently calculated the kappa coefficient. The results are expressed as mean changes with 95% CIs. In all analyses, a two-tailed p < 0.05 was deemed to be statistically significant.

## Results

In total, 88 COPD cases and 45 controls were recruited into the study. The characteristics of the participants are shown in Table 1. The distribution of COPD stages was as follows: GOLD I, 14 patients (15.9%); GOLD II, 41 patients (46.6%); GOLD III, 19 patients (21.6%); and GOLD IV, 14 patients (15.9%). The COPD patients were being treated with inhaled steroids (56.8%), long-acting ß_2 _agonists (55.7%), and tiotropium (50%). The mean dose of inhaled corticosteroids in the COPD group was 844 (377) μg of fluticasone or equivalent per day.

**Table 1:** Baseline characteristics of the study participants

	Entire cohort (n = 133)	COPD (n = 88)	Controls (n = 45)	p value*
Male sex	111 (83.5%)	81 (92%)	30 (66.7%)	< 0.001
Age (years)	62.5 (9.2)	65.4 (7.8)	56.9 (9.2)	< 0.001
BMI (kg/m^2^)	27.6 (5)	27.6 (5)	27.6 (4.9)	NS
Smoking (pack-yr)	56.4 (25.9)	62.2 (25)	45.3 (24.3)	< 0.001
Charlson-age	3.11 (1.5)	3.7 (1.2)	1.8 (1.4)	< 0.001
Exhaled CO (ppm)	8.5 (9.7)	6.5 (7.7)	13.9 (12.4)	0.015
FVC (%)	92.7 (17.4)	92.7 (19)	92.8 (13.8)	NS
FEV_1 _(%)	69.1 (23.4)	58.9 (20.5)	90 (12.8)	< 0.001
Dyspnea (MRC)	0.86 (0.67)	0.97 (0.68)	0.64 (0.6)	0.009

Data expressed as mean (standard deviation) or absolute (relative) frequencies, as appropriate. *Data were analyzed using Student's *t *test or the χ^2 ^test, as appropriate.

FVC: Forced vital capacity. FEV_1_: Forced expiratory volume in 1 second. BMI: body mass index. CO: carbon monoxide. MRC: Medical Research Council scale.

### Concentrations of acute phase reactants

**Figure 1:**
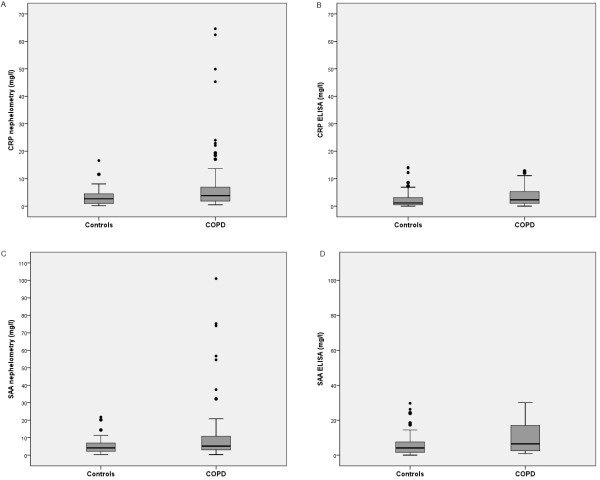
**Concentrations of CRP and SAA measured by the two analytical assays (nephelometry and ELISA) in COPD patients and healthy controls**. Panel a) CRP by nephelometry. Panel b) CRP by ELISA. Panel c) SAA by nephelometry. Panel d) SAA by ELISA.

Compared with controls, the levels of CRP were higher in COPD patients as measured using both assays (CRP (Ne) 7.3 (11.8) vs. 3.3 (3.2) mg/L, p = 0.002; CRP (ELISA) 3.4 (3.2) vs. 2.9 (3.9) mg/L, p = 0.026; Figure 1, Table 2). This difference persisted even after adjustment for age and sex in a multivariable logistic regression model. The adjusted odds ratio for the increased CRP levels (log-transformed) in patients with COPD compared with controls was 3.1 (95% CI: 1.2 to 8.0). The mean value for CRP in the control group was 3.3 mg/L (95% CI: 2.4-4.3 mg/L) for the nephelometric assay and 2.9 mg/L (95%CI: 1.7-4.1 mg/L) for ELISA. Thus, the cut-off value for a normal level was set at 3.1 mg/L. In the COPD group, the mean CRP levels did not differ significantly between smokers and previous smokers (data not shown). Treatment with corticosteroids had no impact on the CRP levels, as assessed by both assays.

**Table 2:** Log-transformed concentrations of CRP and SAA measured by the two analytical assays (nepholometry and ELISA) in COPD patients and healthy controls

	Entire cohort (n = 133)	COPD (n = 88)	Controls (n = 45)	p value*
logCRP (Ne)	0.5 (0.4)	0.6 (0.4)	0.3 (0.4)	0.002
logCRP (ELISA)	0.2 (0.6)	0.3 (0.5)	0.04 (0.7)	0.026
P value†	< 0.001	< 0.001	0.001	

logSAA (Ne)	0.7 (0.4)	0.7 (0.4)	0.5 (0.3)	0.022
logSAA (ELISA)	0.7 (0.4)	0.8 (0.4)	0.5 (0.5)	0.003
P value†	NS	NS	NS	

Data expressed as the mean (standard deviation). *p value calculated by unpaired Student's *t *test. †p value for the difference between nephelometry and ELISA calculated by paired Student's *t *test.

logCRP (Ne): log-transformed concentrations of C-reactive protein measured by nephelometry.

logCRP (ELISA): log-transformed concentrations of C-reactive protein measured by ELISA.

logSAA (Ne): log-transformed concentrations of serum amyloid A measured by nephelometry.

logSAA (ELISA): log-transformed concentrations of serum amyloid A measured by ELISA.

SAA levels measured by ELISA and nephelometry were found to be higher in COPD patients compared with controls (SAA (Ne) 11.0 (17.4) vs. 5.3 (4.7) mg/L, p = 0.022; SAA (ELISA) 9.9 (8.1) vs. 6.6 (7.4) mg/L, p = 0.003; Figure 1, Table 2). This difference persisted even after age and sex were accounted for in a multivariable logistic regression model. The adjusted OR for increased SAA levels (log-transformed) in patients with COPD compared with controls was 2.6 (95% CI: 1.06 to 6.6). The mean value for SAA in the control group was 5.3 mg/L (95% CI: 3.9-6.8 mg/L) for the nephelometric assay and 6.8 mg/L (95% CI: 4.4-9.1 mg/L) for ELISA. Thus, the cut-off value for a normal level was set at 6.05 mg/L. In the COPD group, the mean SAA levels did not differ significantly between smokers compared and previous smokers (data not shown). Treatment with corticosteroids had no impact on SAA levels, as assessed by both assays.

### Comparison between the two assays

The levels of SAA were significantly higher than those of CRP for cases and controls and for both techniques, which indicated a consistently higher expression of SAA (Figure 1, Table 2 p < 0.05 for all comparisons). The levels of CRP (log-scale) correlated moderately with those of SAA (log-scale) as measured by both techniques (Ne: r = 0.54, p < 0.001; ELISA: r = 0.31, p < 0.001). A moderate positive correlation between ELISA and nephelometry measurements was also observed (logCRP: r = 0.55, p < 0.001; logSAA: r = 0.40, p < 0.001). All correlations retained their statistical significance even when the patients and controls were analyzed separately (data not shown).

### Assay agreement

**Figure 2:**
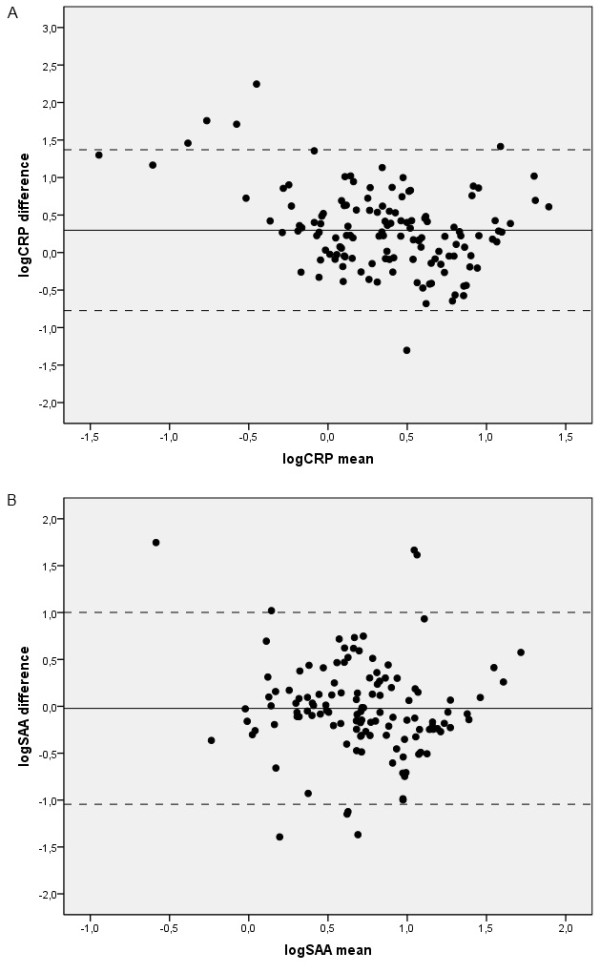
**Bland-Altman plot showing the agreement between CRP and SAA values between the two assays**. Panel a) logCRP. Mean difference: 0.29 (95% CI: -0.77 to 1.37).. Panel b) logSAA. Mean difference: -0.02 (95% CI: -1.04 to 1.0008).

The results of the Bland-Altman plots for nephelometry and ELISA are depicted in Figure 2. There was a consistent difference between the two techniques. Specifically, the values obtained using nephelometry were significantly higher than those obtained using ELISA for both CRP and SAA (Table 3). These differences were even more pronounced when the COPD patients were analyzed separately (Table 3). In the entire sample, the kappa coefficients were 0.34 for CRP (p < 0.001) and 0.21 for SAA (p = 0.015). The patients with COPD showed similar results: specifically, the kappa coefficients were 0.26 (p = 0.007) and 0.23 (p = 0.033) for CRP and SAA, respectively.

**Table 3:** Mean differences in CRP and SAA values measured by the two analytical assays (nepholometry and ELISA) in COPD patients and healthy controls

	Entire cohort (n = 133)	COPD (n = 88)	Controls (n = 45)	p value*
CRP (mg/L)	2.7 (9.4)	3.9 (11.1)	0.4 (4.1)	0.009
SAA (mg/L)	0.31 (14.3)	1.2 (17.2)	-1.4 (5.9)	NS

NS, not significant

* p value calculated by unpaired Student's *t *test.

## Discussion

In this study, results from nephelometry and ELISA were compared in a sample of stable COPD patients. This study provides insight into how assay differences may influence the estimates of inflammation in clinical settings. We are not aware of any other report that has addressed this important clinical and research issue. In fact, the usefulness of systemic biomarkers has been evaluated in several clinical settings (e.g., the emergency room and intensive care unit and during hospitalization) and for a number of diseases; therefore, the standardization of technical assays is necessary for all biomarker studies to provide meaningful data. We were also able to confirm that stable COPD is associated with an increase in CRP and SAA levels compared with healthy controls, which suggests the presence of a systemic inflammatory load, as previously described [3].

Extensive research has investigated the relevance of different inflammatory markers in COPD. CRP and SAA are major acute-phase reactants in humans that are synthesized in the liver by factors that initiate and maintain the inflammatory response, such as interleukin (IL)-1, IL-6, and tumor necrosis factor α. Although several inflammatory mediators induce and control the secretion of these biomarkers, CRP is predominantly induced by IL-6, whereas SAA is stimulated mainly by IL-1 [9].

Increased levels of CRP may reflect the severity of airway inflammation [22] with a strong prognostic value [23], and CRP has recently become a therapeutic target in cardiovascular diseases [24]. Of note, this molecule can provide prognostic information beyond that which is conveyed by the Framingham risk score [25]. However, the exact pathophysiological relevance of high CRP levels remains controversial. A previous study linked CRP to plasma low-density lipoprotein, which may play a significant role in modulating CRP function under physiological conditions [26]. Taken together, these data suggest that CRP may be involved in lipoprotein metabolism, clearance, and deposition.

Increased SAA concentrations have been reported in a number of chronic inflammatory and neoplastic diseases that have been linked to systemic amyloid deposition [27]. Higher serum SAA levels are also associated with COPD exacerbation and are more predictive than CRP for the severity of these episodes [11]. Growing evidence has suggested that SAA may act as an apoprotein. SAA is involved in the conformational change in the high density lipoprotein (HDL) particle [28] and causes increased HDL clearance [29,30]. In light of these findings, it has been hypothesized that increased SAA levels can contribute to accelerated atherosclerosis in stable COPD [13].

Although we found statistically significant method-dependent differences for CRP and SAA, specific differences for each biomarker were noted. First, the distribution of each molecule is noteworthy. As shown in Figure 1, both of the acute phase reactants displayed a wider distribution when measured using nephelometry in COPD compared with the ELISA assays or measurements obtained from the control group. This finding may at least in part explain our results that suggest a more pronounced difference in COPD patients than in controls. This difference is reflected in the standard deviations, which were higher when the biomarkers were measured using nephelometry in the COPD group. Interestingly, this wide distribution has also been reported by other authors who have used different laboratory techniques (e.g., a high sensitivity chemiluminescent immunoassay) [6]. Additionally, the differences between the two techniques were less marked for SAA than for CRP. When the two methods were compared directly (Tables 2 and 3), the differences in SAA levels did not reach the threshold for significance. However, the analysis of agreement showed a consistent difference between the two methods. As shown by the Bland-Altman test, such differences may be clinically important for COPD patients. It is also noteworthy that the kappa coefficient was low.

Despite the high sensitivity, speed, and accuracy as well as the good correlation of nephelometry with other techniques in humans [31,32] and animal models [33], the agreement of this technique with ELISA has not been previously investigated in patients with COPD. Interestingly, a discrepancy between these techniques was reported in an analysis of cerebrospinal fluid IgG [34]. The present study showed that although the concentrations of inflammatory markers obtained using nephelometry and ELISA were moderately correlated, the agreement between the two assays is remarkably low. It is now well-recognized that a good degree of correlation does not necessarily imply a good agreement, and vice versa [21]. Although the two assays correlate well, they may provide different information about COPD patients that could ultimately lead to different conclusions concerning the prognostic significance of the biomarkers.

In this regard, the effect of the assay differences on the selection of appropriate cut-off values for different inflammatory markers is a clinically important issue and therefore must be more precisely characterized. To our knowledge, only one study using nephelometry has attempted to identify a reliable cut-off for increased CRP concentrations (4.21 mg/L) in patients with chronic respiratory disorders [35]. However, other authors have used 3.0 mg/L as a cut-off for high CRP levels that were measured using nephelometry [5]. Although we estimated a cut-off value of 3.1 mg/L for CRP in this study, we found robust evidence of a significant analytical difference between nephelometry and ELISA for the measurement of serum inflammatory markers, with a more marked difference in COPD patients. Although the reasons for the analytical differences between the two assays remain unclear, such a large difference is likely to be clinically significant. It is remarkable in this regard that a difference of 3.9 units for CRP in COPD patients may overcome the threshold value for poor prognostic significance, i.e., 4.21 [35], 3.0 [5] or 3.1 mg/L (the current study).

Although the differences between the assays were less pronounced for SAA, the same reasoning can be applied to this biomarker. The reference values for SAA in healthy individuals have not yet been thoroughly established. To our knowledge, there is only one study on this issue, which identified a threshold value of 7.0 mg/L using an ELISA assay [22]. Of note, this cut-off agrees with that identified in the present study. Using these thresholds, a method-dependent difference of 1.2 mg/L accompanied by a large standard deviation (Table 3) may have a significant impact on the prognostic significance of this marker in a clinical setting. However, a long-term follow-up of the patients would provide insight as to whether these differences in measurements are relevant from a clinical point of view and would determine the variability and reliability of both markers assessed by both methodologies. The methodology used in the present study did not include this follow-up. Future trials should evaluate this aspect in a prospective cohort.

The relationship between inhaled corticosteroid use and the systemic inflammatory load in COPD is a source of debate. In an initial small study, the use of inhaled corticosteroids at a dose of 1,000 μg of fluticasone per day was shown to be effective in reducing serum CRP levels in patients with COPD [36]. However, several years later, the same group found that this association was not present for CRP in a clinical trial multicenter study with a larger sample size [37]. The present study did not show this association, and our results thus provide further evidence for the lack of association.

## Conclusions

In conclusion, the nephelometry values in the present study tended to be higher for both CRP and SAA compared to ELISA measurements in COPD patients. These differences should carefully be considered, as they may actually overcome the commonly accepted threshold values. These findings suggest the presence of a heretofore unrecognized issue in the assessment of systemic inflammation in COPD. International standardization of commercial kits is required before the predictive validity of inflammatory markers for patients with COPD can be effectively assessed in clinical practice. Our results highlight the need for the use of the same analytical procedures when comparing results obtained from different studies. Because standardization is not yet available, studies on inflammatory biomarkers in COPD patients should always include a control group.

## Abbreviations

COPD: chronic obstructive pulmonary disease; ELISA: enzyme-linked immunosorbent assay; CRP: C-reactive protein; SAA: serum amyloid A; FEV_1_: forced expiratory volume in 1 second; FVC: forced vital capacity; ECG: electrocardiogram; Ne: nephelometry; PASW: Predictive Analytics Software; OR: odds ratios; CI: confidence intervals; HDL: high density lipoprotein.

## Competing interests

The authors declare that they have no competing interests.

## Authors' contributions

JLLC was the main responsible for the project, designed the project, included patients, analyzed the data base, performed the statistical computations and wrote the manuscript. EA and AD performed the laboratory determinations. CC included patients in the study and helped with the laboratory determinations. EM and FO included patients in the study. PC performed the clinical tests. FRP and AMW supervised laboratory determinations and the manuscript. All authors read and approved the final manuscript.

## Pre-publication history

The pre-publication history for this paper can be accessed here:

http://www.biomedcentral.com/1471-2288/12/40/prepub
